# Effect of replacing corn silage with alternative silages on quality, fatty acids and volatile compounds of Italian semi-hard raw milk cheese

**DOI:** 10.3389/fvets.2026.1769978

**Published:** 2026-03-09

**Authors:** Sheyla Arango, Nadia Guzzo, Sarah Currò, Emanuele Bianco, Emilio Simonetti, Simona Rainis, Niccolò Renoldi, Nadia Innocente, Lucia Bailoni

**Affiliations:** 1 Department of Comparative Biomedicine and Food Science (BCA), University of Padova, Legnaro, PD, Italy; 2 Regional Agency for the Rural Development (ERSA), Pozzuolo del Friuli, Udine, Italy; 3 Department of Food Science, University of Udine, Udine, Italy

**Keywords:** alternative silages, cheese quality, corn silage, dairy cows, volatile compounds

## Abstract

This study involved 15 lactating cows during three consecutive periods of 21 days each with transition phases of 12 days. Three isonitrogenous and isoenergetic diets (14.5% CP; 38.7% NDF, 22.5% starch, 5.8 MJ/kg DM basis) were used in the first (CS: diet based on corn silage), second (SSS: corn silage was replaced by sorghum-soy silage) and third period (SS: corn silage was replaced by sorghum silage). During the last week of each period, bulk milk from two days was collected to produce six wheels of semi-hard raw milk “Latteria” cheese with 60 days of ripening. Cheese yield was not affected by the diet, averaging 7.03%. Dry matter (60.97% on average) and ash content (3.72% on average) of cheeses showed no significant differences. Cheese from SS had the highest fat content (30.08%), but the lowest protein content (25.29%). Color and physical parameters were significantly affected (*P* < 0.001) by the diet. Cheeses from the SS and SSS diets were brighter and yellower than cheeses from the CS diet. Moreover, cheeses from the SSS and SS diets presented higher values of hardness and cohesiveness and lower elasticity than those from the CS diet. Fatty acid profile was modified by the diet. Cheeses from SSS and SS, compared to CS, contained higher monounsaturated fatty acids (MUFA: 25.11 and 24.75 vs. 21.97% resp.; *P* < 0.001) and lower saturated fatty acids (SFA: 70.61, 70.94 vs. 73.77% resp.; *P* < 0.001). The atherogenic index and thrombogenic index were positively affected (*P* < 0.001) by the alternative crops, as they were higher for CS than for SSS and SS. In conclusion, the use of sorghum and sorghum-soy silages in dairy cow diets modified the color and physical properties of cheese, and improved not only the fat percentage but also the fatty acid profile by increasing monounsaturated fatty acids. For the first time, this study characterized volatile compounds of Latteria cheese and reported that they were affected by the type of silage in the diet.

## 1 Introduction

Dairy cow nutrition is characterized by increased energy and protein demands during the lactation period. Particularly in intensive farming systems, diets typically consist of a total mixed ration (TMR), with corn silage being the widely used feed in the livestock sector for milk production (1–3). Corn silage is predominantly used in dairy ruminants diets due to its high energy content [1.82 Mcal/kg net energy for lactation (NEL)] and adequate protein levels (7%−8% on DM basis) (1, 4).

Despite the excellent nutritional properties of corn silage, its partial or total substitution in farm animal diets has emerged as a necessity to address economic and environmental concerns. Corn cultivation is often associated with negative environmental impacts due to its high irrigation water requirements, fluctuating prices, and potential mycotoxin contamination (1, 5). Consequently, the use of alternative crops, such as autumn-winter cereals or legumes, either individually or in combination, represents a promising alternative for dairy cow's nutrition. After ensiling, these alternative forages can offer good nutritional value, high availability and require less water input during cultivation. Even though sorghum silage is also threatened by fungal diseases, this crop has been studied as an alternative feed ingredient in various farm animals, including dairy cows, buffaloes and goats with encouraging results (5–11). Sorghum silage, with its good protein content, has become an important resource for ruminant feeding, particularly for cattle (10, 12). Similarly, soy silage not only provides a good nutritional composition but also demonstrates high acceptability among dairy cows when it partially replaces corn silage (13, 14).

The use of these alternative crops in dairy cow diets may affect not only milk quality but also cheese characteristics (15). A semi-hard raw-milk cheese called “Latteria” is typically produced in North-Eastern Italy and has not been yet fully characterized in terms of fatty acids and volatile compounds. This Italian cheese is produced through enzymatic coagulation using powdered rennet, followed by curd heating to 44 °C−48 °C and a minimum ripening period of 60 days. Recent regulations in the official manufacturing procedure of Italian cheeses, have introduced the use of cereal silages to reduce the risk of aflatoxin contamination associated with the traditional corn silage (16). Consequently, the evaluation of additional cereal species or alternative crops aims to identify feeding strategies that support both cheese quality and food safety.

Several studies have shown that using sorghum silage can lead to qualitative improvements in cheese, particularly through a significant increase in fat content (9, 10, 17). For instance, a Mexican fresh cheese called “Molido” showed increased fat and protein contents when 80% of corn silage was replaced with sorghum silage in Holstein cows' diets (9). Similar effects were observed in goat cheese, with fat content increasing as sorghum silage was progressively included in the diet (10). Moreover, Mozzarella cheese maintained its quality when sorghum silage replaced corn silage in buffalo diets (17). These results 9 versatility of sorghum silage across various dairy species and cheese types, reinforcing its potential as a viable alternative to corn silage in line with the United Nations Sustainable Development Goal 2: Zero Hunger (18). Therefore, this study aimed to evaluate the effects of substituting corn silage with either a mixture of sorghum-soy silage or sorghum silage on the physicochemical properties, fatty acid profile, and volatile profile of the “Latteria” semi-hard raw-milk cheese.

## 2 Materials and methods

This study was performed in accordance with the ethical committee of the University of Padova (approval number 61/2025) and carried out according to the directive 2010/63/UE of the European Parliament on the protection of animals used for scientific purposes and the Italian law on animal care (Legislative Decree No. 26 of 14 March 2014).

### 2.1 Animals and experimental design

The study was conducted at the dairy farm of the Institute of Higher Education ISIS Paolino d'Aquileia located in Cividale del Friuli, Udine, Italy. The farm consisted of a free-stall barn equipped with an automatic milking system (Dairy Robot R9500, GEA). The entire herd of the farm composed of fifteen Italian Simmental lactating cows (DIM: 149 ± 67; Parity: 2.6 ± 1.4; Milk yield: 25.7 ± 4.6 kg/d) was involved in the study. The trial was divided into three consecutive experimental periods corresponding to three experimental diets (CS: corn silage, SSS: sorghum-soy silage, and SS: sorghum silage). The duration of each period was of 21 days. To pass from one period to another, transition periods of 12 days of duration were considered in order to gradually change the diet.

### 2.2 Silages and diet

The corn silage used came from the same vendor located at the Friuli Venezia Giulia Region (UD, Italy) and was baled using a stationary round baler to ensure a uniform batch for the whole experiment. The two fields used to produce the alternative crops silages were located at Cividale del Friuli and Pozzuolo del Friuli, Udine, Italy. They both followed the same agronomical and silage procedure. All forages were sown mechanically on June, 2023 and mown in September, 2023 using a disc mower conditioner. For the sorghum silage, the hybrid seeds PR845F (*Sorghum bicolor x Sorghum bicolor*) were used at a density of 40 kg/ha. For the sorghum-soy silage, the sorghum hybrid seeds Nicol (*Sorghum bicolor x Sorghum sudangrass*) and the Adonai variety soy were sown together at a density of 40 and 85 kg/ha respectively. Forages were transported into the farm, and a trench silo was assigned for each silage. A bacterial inoculant Pioneer 11GFT (Corteva Agriscience, Indiana, US) based on *Lactobacillus buchneri* LN40177 was added before the completion of 6 months of fermentation process. The silage was cut once daily from the trench silo.

In accordance with the guidelines outlined by the National Research Council (19), all animals were fed using three isonitrogenous and isoenergetic diets (14.5% CP; 38.7% NDF, 22.5% starch, 5.81MJ/kg DM basis): a diet with corn silage (CS) being replaced by sorghum-soy silage (SSS), and sorghum silage (SS) to fulfill energy requirements of lactating dairy cows. The diet provided as a total mixed ration (TMR) was distributed twice a day during the morning (8:30 a.m.) and the afternoon (5:30 p.m.). Daily intake was not recorded individually but could be estimated from the daily feed distribution resulting in 21.7, 24.6 and 17.0 kg of DM for the CS, SSS and SS diets respectively. Fresh water was available *ad libitum*.

Silage (Table 1) and diet (Table 2) samples were collected at the beginning and the end of the experiment, and then analyzed for nutritional composition using Near-Infrared Spectroscopy (NIRs) with a DS 2500 FOSS instrument (Foss Analytical, Hileroed, Denmark; spectral range 850–2,500 nm, reflectance mode). Corn silage showed higher DM than the alternative silages. The three silages were similar in crude protein and fat content. Concentrations of starch and non-structural carbohydrates (NSC) were much greater in corn silage, whereas NDF and ADF were greater in the alternative silages in agreement with NRC (19) tables. The silage making process could be considered optimal for all the silages as suggested by the pH values, the small proportion of propionic and butyric acids, and the much higher production of lactic than acetic acid.

**Table 1:** Composition (% DM) of the silages used in the experimental diets.

**Item**	**Corn silage**	**Sorghum-soy silage**	**Sorghum silage**
Dry Matter, %	29.99	25.77	24.73
Ash	4.05	8.30	7.85
Crude protein	7.44	7.99	7.70
Fat	2.91	2.49	2.78
Starch	33.17	5.76	11.15
NSC	45.15	18.59	24.20
NDF	40.46	62.63	57.47
ADF	22.42	36.69	32.95
AIA	0.36	0.08	0.31
pH	3.76	4.26	4.26
NH_3_-N (%N tot.)	7.51	8.16	8.41
Lactic acid	5.11	2.47	2.64
Acetic acid	2.06	2.41	2.05
Propionic acid	0.56	0.19	0.21
Butyric acid	0.10	0.24	0.28
UFL	0.88	0.72	0.75

DM, dry matter; NSC, non-structural carbohydrates; NDF, neutral detergent fiber; ADF, acid detergent fiber; AIA, acid insoluble ashes; NH3-N, ammoniacal nitrogen; UFL, forrage unit for lactation.

**Table 2:** Ingredients and chemical analysis of the rations fed in the experimental diets.

**Ingredients (kg As fed)**	**CS**	**SSS**	**SS**
Corn silage	15.0	–	–
Sorghum-soy silage	–	15.0	5.0
Sorghum silage	–	–	16.0
Mile hay	–	3.0	–
Meadow hay	3.0	–	–
Alfalfa hay	7.5	6.0	7.5
Corn Meal	2.0	3.5	3.0
Wheat Meal	1.0	1.5	2.0
Premix^a^	1.0	1.2	1.0
Soybean meal	0.5	0.5	0.5
Sodium bicarbonate	0.1	0.1	0.1
Water	4.0	2.0	0
Compound feed^b^	2.0	2.0	2.0
Total	36.1	34.8	37.1
**Chemical composition, % of DM**			
DM, % as fed	50.53	52.45	40.80
Crude Protein	14.70	14.93	13.98
Lipids	2.56	2.78	2.38
NDF	35.81	37.76	42.45
ADF	22.95	25.05	26.75
AIA	0.81	0.69	0.78
Starch	23.02	22.61	21.92
NE_L_, MJ/kg DM	5.82	5.89	5.72

^a^Chemical composition of premix (%DM): Moisture 10.95%, Crude Protein 33.86, Lipids 2.540, Ash 13.817.40, NDF 21.87.

^b^Chemical composition of compound feed (%DM): Moisture 13.00%, Crude Protein 18.50, Lipids 3.20, Crude Fiber 6.10, Sodium 0.43, Ash 7.40.

CS, corn silage diet; SSS, sorghum-soy silage diet; SS, sorghum silage diet; DM, dry matter; NDF, neutral detergent fiber; ADF, acid detergent fiber; AIA, acid insoluble ashes; NE_L_, Net energy of lactation.

### 2.3 Milk, cheese making and analysis

Cheese making took place at the pilot milk laboratory of the ISIS Paolino d'Aquileia using the daily milk production of the entire herd. The chemical composition of the milk coming from the CS (3.2% fat, 3.6% protein and 4.6% lactose), SSS (3.9% fat, 3.5% protein and 4.6% lactose) and SS (3.5% fat, 3.6% protein and 4.6% lactose) diet was taken from the automatic milking machine (Dairy Robot R9500, GEA). For each experimental period (diet), cheeses were manufactured two times (2 days of the last week of the diet sumministration). On each day of manufacturing, a total of three cheeses were obtained according to the traditional semi-hard cheese technology parameters.

Milk processing was performed following the traditional production techniques laid down for the Latteria cheese with small modifications (20). Initially, raw milk of the day was weighed (CS: 504.7 and 556.5 kg; SSS: 587.1 and 618 kg; SS: 618 and 597.4 kg). Then, milk was subjected to pH (6.9–7.0) measurements before being heated to 37 °C under continuous agitation. Selected starter cultures (TNSD Lyboac-D, containing *Streptococcus salivarius* subsp. *thermophilus*) were added at a standardized dosage of 10^6^ per 400–500 L, followed by further pH (6.4–6.6) monitoring. Coagulation was induced by adding powdered rennet (1,000 IMCU/g, 95% chymosin) at a dosage of approximately 4 g/hL, maintaining the milk at 36 °C for 35 min. The curd was then mechanically cut in three phases: first into 10 x 10 mm cubes, then reduced to hazelnut-sized particles, and finally to rice grain size at 36 °C−37 °C over 20 min. Cooking followed, with gradual heating to 44 °C−45 °C while maintaining agitation for 20 min. After a short resting phase, whey was partially drained, and the curd was transferred for molding and whey expulsion. The pH was taken before (5.2–5.4) and after (5.1–5.3) the cheese went into progressive pressing cycles (2 atm for 25–30 min, 3 atm for 25–30 min, and a final 3 atm press for 24 h). Salting was performed in a 20% brine solution for 24 h. The cheeses were then stored in a ripening chamber at 9 °C with 85% relative humidity for 60 days, ensuring the biochemical and microbial transformations required for flavor and texture development. Finally, cheese yield was calculated post-pressing and considered as a technological parameter to assess production efficiency. Overall, 18 cheeses were produced in total, six for each dietary treatment.

The physicochemical analyses were carried out at the La. Chi. Laboratory of the University of Padova, Italy. Cheeses were analyzed at 60 days of ripening in triplicate. The determination of moisture, ash, lipids and crude protein content (*N* ^*^ 6.38) were performed following standard methods (21). The determination of the instrumental color was carried out using a Minolta spectrophotometer (CM-600d, Konica Minolta Inc, Tokyo, Japan) through the CIELAB system defined by L^*^, a^*^ and b^*^ corresponding to lightness, redness and yellowness, respectively. Rheological parameters were determined by a texture profile analysis (TPA) using the Texture Analyzer HDI dynamometer (Stable Micro Systems Ltd., England) and calculated by the Texture Exponent Connect software (Vers. 4.0.9.0; Stable Micro Systems Ltd., England). Measurements were made at 20 °C ± 1 °C. TPA was applied to the cylinders of cheese (70 x 40 x 20 mm) compressed axially in two consecutive cycles, with a deformation of 30% of the original height and applying a force of 60 mm/min. Three parameters were determined: hardness, cohesiveness and elasticity.

Cheese samples were analyzed at 60 days of ripening for the determination of their fatty acid (FA) profile. Lipid extraction was done according to the ISO Methodology 14156 (22). Esterification and the detection of the fatty acid methyl esters (FAMEs) were described in detail in the procedure of Bittante et al. (23) and were evaluated using a gas chromatograph with flame-ionization detector (Agilent Technologies Inc., Shangai, China). The identified fatty acids were reported as a mean relative percentage of the total FAME.

### 2.4 Determination of volatile compounds

The determination of volatile compounds in the headspace of cheese samples was carried out using a solid-phase microextraction (SPME) coupled to the gas chromatography-mass spectrometry (GC–MS, QP2020 NX, Shimadzu Corporation, Kyoto, Japan) instrument, as previously described by Renoldi et al. (24). Briefly, a 5 g sample of ground cheese was weighed in a 20 ml vial and sealed with silicone septum and aluminum cap. Sample equilibration was conducted at 60 °C for 30 min with an HT2800T autosampler (HTA s.r.l., Brescia, Italy). For the extraction step, a 2-cm × 50/30-μm Stableflex 24 Ga divinylbenzene/carboxen/poly-dimethylsiloxane (DVB/CAR/PDMS) coated SPME fiber (Supelco, Bellefonte, PA, USA) was used. Volatile compounds separation was performed using a DB-WAX capillary column (30 m length, 0.25 mm internal diameter, and 0.25 μm film thickness; Agilent Technologies, CA, USA), helium flow rate, and a temperature ramp. The analyses were performed in scan mode over a mass range of 25–350 m/z. Chromatographic data were processed using GC–MS Solution software version 4.52 (Shimadzu Corporation, Kyoto, Japan). Compound identification was carried out by comparing the acquired spectra with those of commercial standards (2-heptanone, 2-nonanone, 3-methylbutanoic acid, butanoic acid, and hexanoic acid; Sigma Aldrich, Italy), the NIST/EPA/NIH 20 Mass Spectral Library (John Wiley & Sons Inc., Hoboken, NJ, USA), and with retention indices (RI) reported in the literature (https://webbook.nist.gov/chemistry/). Quantification was based on the absolute peak areas obtained from the headspace of each sample.

### 2.5 Statistical analysis

Cheese quality parameters were analyzed using a completely randomized design with three treatments (diets: CS, SSS, and SS). To verify the difference among treatments, an analysis of variance (ANOVA) was performed, and then a Tukey test was applied at a level of 5% probability using the SAS Institute Software (SAS Institute Inc., Cary NC, USA, 2014). For the volatile compound profile, Principal Component Analysis (PCA) was performed using the Origin Pro 9 software (OriginLab, Northampton, USA).

## 3 Results

Table 3 reports the effect of the dietary treatment on yield and physicochemical composition of cheeses.

**Table 3:** Cheese yield and physicochemical composition.

**Parameter**	**CS**	**SSS**	**SS**	**SEM**	***P*-value**
Yield post-pressing, %	9.74	9.49	9.09	0.1127	0.2396
Yield after 60 days, %	7.61	6.96	6.42	0.4901	0.3628
pH	5.32^b^	5.49^ab^	5.51^a^	0.0129	0.0209
**Chemical composition**					
DM, %	60.53	62.09	60.28	1.5704	0.0500
Ash, %	4.15	3.56	3.57	0.2711	0.1141
Protein, %	26.70^A^	25.29^B^	26.02^AB^	0.4676	0.0096
Fat, %	25.18^C^	30.08^A^	28.16^B^	0.8585	< 0.0001
**Color parameters**					
L^*^, lightness	78.56^B^	80.65^A^	81.66^A^	0.8317	0.0001
a^*^, redness	−0.14^B^	0.23^A^	−0.14^B^	0.0170	0.0002
b^*^, yellowness	16.27^B^	17.42^A^	17.63^A^	0.3575	0.0027
**Physical parameters**					
Hardness, *N*	37.69^B^	70.84^A^	62.74^A^	67.0088	< 0.0001
Cohesiveness	0.69^B^	0.76^A^	0.76^A^	0.0006	0.0002
Elasticity, mm	0.99^A^	0.64^B^	0.59^B^	0.0461	0.0098

CS, corn silage diet; SSS, sorghum-soy silage diet; SS, sorghum silage diet; SEM, standard error.

^a, b, ab^Differences between diets (*P* < 0.05).

^A, B, AB^ Differences between diets (*P* < 0.01).

### 3.1 Cheese yield and chemical composition

Cheese yield after 60 days of ripening was not affected by the diet and was in average 6.99%. The pH of cheese was highest for the SS diet (5.51; *P* < 0.05). Regarding the chemical composition, there were significant differences for fat and protein content. In particular, diets using alternative silages (SSS and SS) produced cheeses higher in fat and lower in protein content than the ones based on CS. The fat content of cheeses from SSS resulted higher than SS and CS (30.08, 28.16 and 25.18% resp.; *P* < 0.001). On the contrary, SSS showed the lowest protein content in comparison to CS and SS (25.29 vs. 26.70 and 26.02% resp.; *P* < 0.01). The ash content showed no difference between treatments with an average value of 3.76%.

### 3.2 Color and physical parameters of cheese

Pairwise comparisons showed significant differences among dietary treatments as cheeses differed in color. The CS diet produced a less bright cheese than the other type of crops (SSS and SS) (L^*^: 78.56, 80.65 and 81.66 resp.; *P* < 0.001). The SSS diet produced cheese with a higher redness than the diets based on CS and SS silage (a^*^:0.23, −0.14 and −0.14 resp.; *P* < 0.001). Cheeses coming from diets using alternative crops (SSS and SS) showed significantly higher yellowness than the CS diet (b^*^: 17.42, 17.63 and 16.27 resp.; *P* < 0.01).

The physical parameters of cheeses showed significant differences between diets. Moisture content was comparable among treatments, indicating similar aqueous phase conditions within the cheese matrix; therefore, the observed differences in textural parameters are likely attributable to the diet or to factors other than serum phase variability. Compared to CS, cheeses based on diets using alternative crops (SSS and SS) presented higher values of hardness (37.69, 70.84 and 62.74 N resp.; *P* < 0.001) and cohesiveness (0.69, 0.76 and 0.76 resp.; *P* < 0.001); therefore, these groups also presented significantly lower values of elasticity (0.99, 0.64 and 0.59 mm resp.; *P* < 0.01).

### 3.3 Fatty acid composition of cheese

The complete fatty acid profile of the cheeses is shown in Table 4. Significant changes were observed in 50 FAs (*P* < 0.05) due to the diet. The sum of SFA was higher for cheeses coming from the CS diet in comparison to SSS and SS (73.77, 70.61 and 70.94% resp.; *P* < 0.001). Among these, palmitic acid (C16:0) was the most the abundant and resulted higher for the CS diet than with the alternative forages SSS and SS (36.67, 35.65 and 35.85% resp.; *P* < 0.01). Myristic acid (C14:0), the second most abundant, was also higher for the CS diet than with the other two SSS and SS (13.50, 12.13, 12.68% resp.; *P* < 0.001).

**Table 4:** Fatty acid (%FAME) composition of cheese.

**Fatty acid**	**CS**	**SSS**	**SS**	**SEM**	***P*-value**
C4:0	1.54^A^	1.29^B^	1.19^C^	0.002	< 0.0001
C5:0	0.04^A^	0.02^B^	0.02^B^	0.000	< 0.0001
C6:0	1.65^A^	1.33^B^	1.28^B^	0.002	< 0.0001
C7:0	0.04^A^	0.03^B^	0.03^B^	0.000	< 0.0001
C8:0	1.25^A^	1.01^B^	1.01^B^	0.001	< 0.0001
C9:0	0.06^A^	0.05^B^	0.06^A^	0.000	0.0130
C10:0	3.36^A^	2.71^B^	2.84^B^	0.010	< 0.0001
C11:0	0.12^A^	0.07^C^	0.09^B^	0.000	< 0.0001
C12:0	4.27^A^	3.48^C^	3.73^B^	0.159	< 0.0001
C13:0 iso	0.04^B^	0.07^A^	0.07^A^	0.000	< 0.0001
C13:0 anteiso	0.08	0.08	0.08	0.000	0.0554
C13:0	0.18^A^	0.13^C^	0.15^B^	0.000	< 0.0001
C14:0 iso	0.12	0.13	0.13	0.000	0.0608
C14:0	13.50^A^	12.13^C^	12.68^B^	0.057	< 0.0001
C15:0 iso	0.26^b^	0.27^a^	0.26^b^	0.000	0.0416
C15:0 anteiso	0.55^A^	0.55^A^	0.51^B^	0.000	0.0112
C15:0	1.57^A^	1.33^C^	1.43^B^	0.000	< 0.0001
C16:0	36.67^A^	35.65^B^	35.85^B^	0.234	0.0055
C17:iso	0.02^B^	0.04^A^	0.05^A^	0.000	< 0.0001
C17:0	0.63^B^	0.77^A^	0.74^A^	0.001	< 0.0001
C18:0 iso	0.08^A^	0.01^B^	0.01^B^	0.000	< 0.0001
C18:0	7.60^C^	9.20^A^	8.47^B^	0.186	< 0.0001
C20:0	0.10^B^	0.21^A^	0.19^A^	0.000	< 0.0001
C21:0	0.04^B^	0.06^A^	0.05^A^	0.000	0.001
C10:1	0.27^A^	0.22^B^	0.23^B^	0.000	< 0.0001
C14:1cis9	0.89^A^	0.72^B^	0.77^B^	0.001	< 0.0001
C15:1trans10	0.22^A^	0.01^B^	0.01^B^	0.000	< 0.0001
C16:1 trans9	0.04^C^	0.37^A^	0.32^B^	0.000	< 0.0001
C16:1 n9 cis7	0.24^A^	0.21^B^	0.20^B^	0.000	0.005
C16:1 n7 cis9	1.15	1.21	1.25	0.017	0.3866
C16:1 n6 cis10	0.50^B^	0.57^AB^	0.65^A^	0.003	0.0024
C16:1 n5 cis11	0.03^B^	0.05^A^	0.03^B^	0.000	0.0039
C16:1 n4 cis12	0.01^B^	0.02^AB^	0.03^A^	0.000	0.0048
C17:1 n5 trans11	0.01^C^	0.03^B^	0.04^A^	0.000	< 0.0001
C17:1 n8 cis9	0.24	0.25	0.23	0.000	0.0644
C18:1 n10 trans 6/8	0.09^B^	0.13^A^	0.15^A^	0.000	< 0.0001
C18:1 n9 trans9	0.08^A^	0.06^B^	0.07^B^	0.000	0.0029
C18:1 n8 trans10	0.23^A^	0.10^B^	0.11^B^	0.000	< 0.0001
C18:1 n7 trans11	0.48	0.48	0.43	0.003	0.2639
C18:1 n12 cis6	0.16	0.17	0.21	0.002	0.1734
C18:1 n9 cis9 (+trans 15)	15.89^B^	19.13^A^	18.54^A^	0.442	< 0.0001
C18:1 n7 cis11	0.78	0.62	0.73	0.016	0.0924
C18:1 n6 cis12	0.32^a^	0.22^b^	0.22^b^	0.004	0.0376
C18:1 n5 cis13	0.04^B^	0.06^A^	0.05^A^	0.000	0.0029
C18:1 n12 trans16	0.16	0.19	0.20	0.002	0.1676
C18:1 n4 cis15	0.06^C^	0.12^A^	0.11^B^	0.000	< 0.0001
C20:1 n9 cis11	0.05^B^	0.14^A^	0.14^A^	0.000	< 0.0001
C22:1 n9 cis13	0.02^B^	0.03^A^	0.03^A^	0.000	0.0041
C18:2 cis9 trans13	0.12	0.12	0.13	0.000	0.0680
C18:2 n3 trans11 cis15	0.05^B^	0.08^A^	0.05^B^	0.000	< 0.0001
C18:3 n3 cis 9,12,15 alpha	0.58	0.54	0.52	0.003	0.1861
C18:2 cis9 trans11 CLA	0.27^B^	0.27^B^	0.29^A^	0.000	0.0076
C20:3 n3 cis 11,14,17	0.03	0.03	0.02	0.000	0.6570
C20:5 n3 EPA	0.07^a^	0.06^b^	0.06^b^	0.000	0.0377
C21:5 n3	0.01^B^	0.09^A^	0.09^A^	0.000	< 0.0001
C22:5 n3	0.11^B^	0.13^A^	0.12^B^	0.000	0.0045
C22:6 n3 DHA	0.04	0.01	0.02	0.001	0.2559
C18:2 n6 8trans 12cis	0.11^B^	0.16^A^	0.16^A^	0.000	< 0.0001
C18:2 n6 cis9 trans12	0.12^A^	0.03^B^	0.04^B^	0.000	< 0.0001
C18:2 n6 cis 9 cis12	2.28	2.28	2.32	0.013	0.7803
C18:3 n6 cis 6,9,12	0.04	0.04	0.04	0.000	0.3911
C20:2 n6 cis 11,14	0.04^C^	0.08^B^	0.10^A^	0.000	< 0.0001
C20:3 n6 cis 8,11,14	0.12	0.13	0.13	0.000	0.8209
C20:4 n6 cis 5,8,11,14	0.20	0.19	0.18	0.000	0.0432
C22:2 n6 cis 13,16	0.02^A^	0.01^B^	0.01^B^	0.000	0.0001
C22:4 n6	0.03	0.04	0.04	0.000	0.1519
C22:5 n6	0.01	0.01	0.01	0.000	0.2770
SFA	73.77^A^	70.61^B^	70.94^B^	0.894	< 0.0001
MUFA	21.97^B^	25.11^A^	24.75^A^	0.681	< 0.0001
PUFA	4.26	4.28	4.31	0.070	0.8863
Σn3	1.28	1.32	1.29	0.005	0.5628
Σn6	3.80	3.75	3.90	0.021	0.2435
n6/n3	2.98	2.84	3.02	0.019	0.0903
AI	3.62^A^	2.98^B^	3.12^B^	0.021	< 0.0001
TI	3.39^A^	3.04^B^	3.09^B^	0.017	0.0006

CS, corn silage diet; SSS, sorghum-soy silage diet; SS, sorghum silage diet; SFA, saturated fatty acids; MUFA, monounsaturated fatty acids; PUFA, polyunsaturated fatty acids; SEM, standard error. AI, atherogenic index [C12:0 + (4 × C14:0) + C16:0]/(MUFA + PUFA); TI, thrombogenic index [C14:0 + C16:0 + C18:0]/[(0.5 × MUFA) + (0.5 × Σn6) + (3 × Σn3) + (Σn3/Σn6)].

^a, b, ab^Differences between diets (*P* < 0.05).

^A, B, AB^Differences between diets (*P* < 0.01).

The greatest proportion of total MUFA (*P* < 0.001) were found in the cheeses from SSS (25.11%) and SS (24.75%). The major FA from this group was oleic acid (C18:1 cis-9), which was higher for cheeses coming from the SSS and SS diets than with the CS diet (19.13, 18.54 and 15.89% resp.; *P* < 0.001).

Regarding the proportion of PUFA, there were no significant differences between cheeses coming from the different diets tested in this trial and the average was 4.29%. Most of the n3 FAs in cheese belonged to α-linolenic acid (C18:3 cis-9, 12, 15) with a mean of 0.55%. Rumenic acid (CLA 18:2 cis9 trans11) was higher in cheeses from the SS diet than the CS and the SSS diet (0.29, 0.27 and 0.27 resp.; *P* < 0.01). The rest of the n3 FAs were detected in very low amounts. Nor the sum of n3 FAs (3.82) or the sum of n6 FAs (1.30) were affected by the dietary treatment.

Regarding the n6:n3 ratio, cheeses were not affected by the diet showing an average value of 2.95. Meanwhile, the atherogenic (AI) and the thrombogenic index (TI) of the cheeses were positively affected by the use of alternative crops in the diet. In fact, the atherogenic index resulted higher for cheeses from CS than SSS and SS (3.62, 2.98 and 3.12 resp.; *P* < 0.001). The same was found for the thrombogenic index, which was higher for cheeses from CS than SSS and SS (3.39, 3.04 and 3.09 resp., *P* < 0.001).

### 3.4 Cheese volatile profile

**Figure 1:**
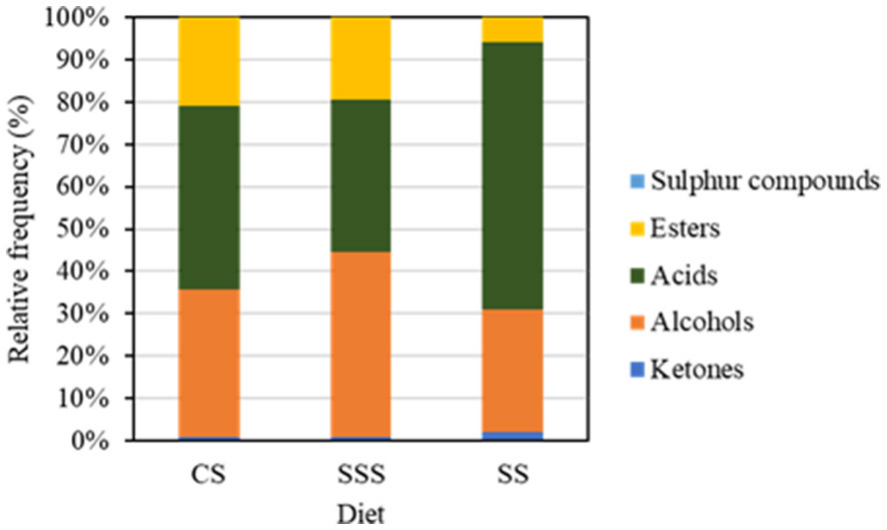
Relative frequency distribution of aromatic classes (acids, alcohols, ketones, esters, and sulfur compounds) of the experimental cheeses. CS, corn silage diet; SSS, sorghum-soy silage diet; SS, sorghum silage diet.

Table 5 lists all the components identified in cheeses. Overall, a total of 40 volatile compounds were identified. From these compounds, 2 propanol, 2 pentanone and 2 nonanone were not found in the CS and SSS cheeses. Meanwhile, pentanoic acid hexanoic acid was not found in the SS cheese. Figure 1 shows the distribution of volatile compound classes (acids, alcohols, esters, ketones and sulfur compounds). The two largest chemical families in terms of relative quantity were acids and alcohols. Dimethyl sulfone was the only sulfur compound detected at low levels in all of the cheeses.

**Table 5:** Volatile compounds of cheese.

**RI**	**Chemical compound**	***m*/*z***
887	Ethyl Acetate	61, 29
903	2-Butanone	43, 72
923	2-Propanol	45, 27
936	Ethanol	31, 49
978	2-Pentanone	43, 86
1,028	2-Butanol	45, 59
1,046	Butanoic acid, ethyl ester	71, 88
1,107	1-Propanol, 2-methyl-	27, 43
1,141	2-Pentanol	55, 45
1,149	Pentanoic acid, ethyl ester	29, 85
1,163	1-Butanol	56, 31
1,181	2-Heptanone	71, 58
1,217	1-Butanol, 3-methyl	42, 70
1,243	Hexanoic acid, ethyl ester	88, 29
1,289	Acetoin	45, 88
1,325	2-Heptanol	45, 73
1,342	Heptanoic acid, ethyl ester	88, 113
1,359	1-Hexanol	29, 57
1,389	2-Nonanone	43, 71
1,438	Octanoic acid, ethyl ester	88, 127
1,445	Acetic acid	60, 43
1,537	Propanoic acid	28, 74
1,541	2,3-Butanediol, [R]	45, 29
1,560	1-Octanol	56, 69
1,568	Propanoic acid, 2-methyl	56, 84
1,578	2,3-Butanediol [S]	73, 43
1,627	Butanoic acid	60, 73
1,645	Decanoic acid, ethyl ester	88, 101
1,671	Butanoic acid, 3-methyl-	43, 60
1,738	Pentanoic acid	73, 45
1,776	1,3-Propanediol	28, 31
1,845	Hexanoic acid	60, 73
1,901	Dimethyl sulfone	57, 71
1,917	Phenylethyl Alcohol	91, 122
1,953	Heptanoic acid	73, 41
2,060	Octanoic acid	43, 73
2,168	Nonanoic acid	41, 60
2,274	n-Decanoic acid	73, 60
2,339	9-Decenoic acid	55, 69
2,486	Dodecanoic acid	60, 73

RI, Kovats retention index.

m/z, mass to charge ratio.

**Figure 2:**
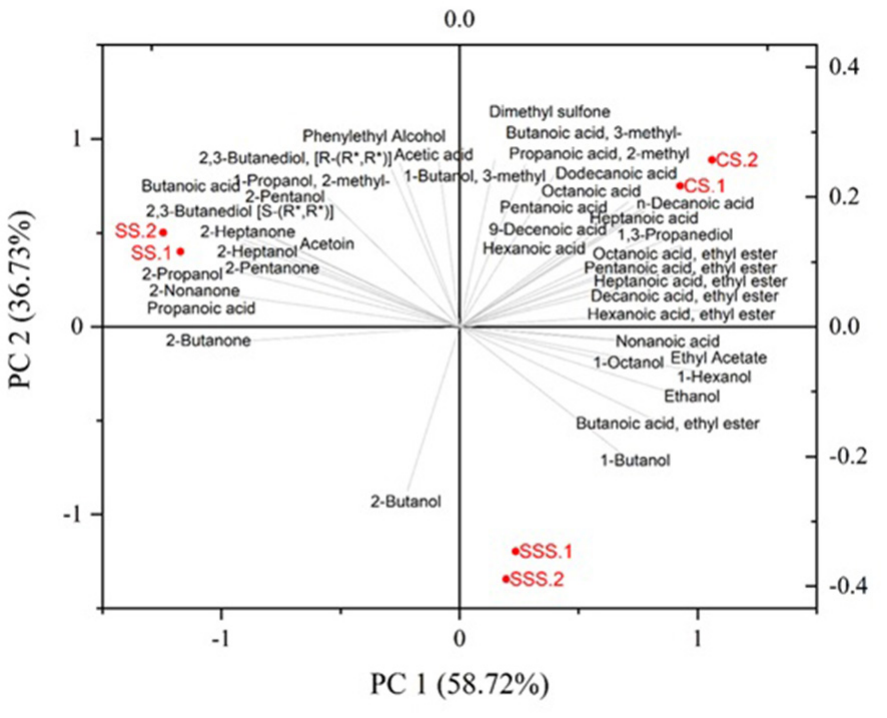
Principal component analysis (PCA) biplot of volatile compounds of the experimental cheeses. CS, corn silage diet; SSS, sorghum-soy silage diet; SS, sorghum silage diet, replicates 1 and 2.

As displayed in the PCA biplot (Figure 2), the first principal component (PC1) accounted for 58.72% of the overall data variability, whereas the second component (PC2) contributed to an additional 36.73%, resulting in a cumulative explained variance of 95.45%. The three experimental cheeses were located in different quadrants of the PCA biplot, indicating notable differences in volatile compounds produced after the 60 days of ripening.

## 4 Discussion

The traditional semi-hard raw-milk cheese produced in North-Eastern Italy, typically characterized by a ripening period of about 60 days, is commonly known as Latteria cheese. As the production of this cheese has not yet been standardized, only a few studies are available in the literature for meaningful comparison (25, 26). Therefore, the results presented here may contribute to the characterization of this traditional Italian cheese.

### 4.1 Cheese yield and chemical composition

On this experiment, cheese yield was not affected by the silage replacement in the diet. Similarly, a study reported no differences on Mozzarella cheese yield coming from lactating buffaloes fed diets based on corn silage or sorghum silage (17). Moreover, no effect on yield was found in Parmigiano Reggiano cheeses produced from cows fed a hay-based diet replacing the whole amount of corn meal with sorghum meal (27).

The chemical composition of the Latteria cheese produced in this experiment was similar to the only scientific article found in the literature for the same type of cheese. Particularly, a study that gathered 79 samples of an Italian semi-hard cheese reported mean values for moisture, fat and protein content of 41.8%, 29.9% and 22.8% respectively (25). In accordance with some findings reported in the literature, the chemical composition of cheese was modified by the use sorghum silage in the diet (9, 10, 28). Replacing corn silage with sorghum and sorghum-soy silages in the diet of dairy cows, resulted in a decrease of protein but at the same time in an increase of fat content of the cheese considered. The higher protein concentration in cheese coming from the CS diet could be associated with the higher starch of the corn silage. Meanwhile, the higher fat content in cheese coming from the alternative silages could be related to the greater NDF and the greater use of corn meal (as a starch source) provided by the SSS and SS diets. The same effect for fat content was also found in a study comparing the chemical composition of fresh “molido” cheese made with milk coming from cows fed with 100% corn silage or 20% corn silage + 80% sorghum silage, which authors attributed to the higher tannin content of sorghum (9). Also, goat cheese coming from different levels of sorghum silage inclusion (7.5, 15, 30 and 45% DM) in the diet, increased gradually their fat content and decreased their protein content (10).

### 4.2 Color and physical parameters of cheese

The color traits of the semi-hard raw-milk cheese were similar to the only study of characterization of this type of cheese which reported values for brightness, redness and yellowness of 79.5, −0.9 and 14.1 respectively (25). Similarly, colorimetric parameters of Montasio cheese at 60 days of ripeness were comparable with Latteria cheese in this study (28). As regards the effect of the treatment, the diet based on corn silage (CS) had the lowest values for all the color parameters: brightness, redness and yellowness. Which agrees with the literature that reported that the color of cheese varies with the diet (28–30), especially when they are based in grass with a high content of carotenoids.

The diet affected the rheological characteristics of the cheese. Texture of cheese depends on its chemical composition, mostly due to the water content (28). In fact, the sorghum-soy silage diet (SSS) that produced the cheese with the lowest water content (37.91%) showed the highest value for hardness (70.84 N). Meanwhile, the cheese coming from the sorghum silage diet (SS) had a similar value of hardness (62.74 N) as the Montasio cheese (62.8 N) at 60 days of ripeness (28). Even though no significant differences were found in cohesiveness of mozzarella cheese coming from buffalo milk based on a corn silage diet (0.52) and a sorghum silage diet (0.51) (31). Our study found that cohesiveness was affected by the diet, and cheeses coming from diets based on sorghum-soy (SSS: 0.76) and sorghum silage (SS: 0.76) had higher values than the corn silage diet (CS: 0.69).

### 4.3 Fatty acid composition of cheese

Results were in accordance to the reported values for Latteria cheese from the Italian Veneto Region with values of 67.15, 28.21 and 4.64 % for SFA, MUFA and PUFA respectively (23). As stated in the literature, diet can modify the FA profile of cheese (30, 32, 33). Cheese is known to contain high proportions of SFA, which is considered to be negative for the consumers' health. When replacing corn silage, alternative crops silages had clear effects on improving cheese FA profile as they increased MUFA and decreased SFA.

The use of sorghum silage and sorghum-soy silage as ingredients in dairy cows' diets reduced the concentration of some saturated fatty acids (C12:0, C14:0 and C16:0) in cheese that are known to have negative consequences in human health because they are hypercholesterolemic (34). In this study, the two diets using the alternative silages differed to the CS diet on the NDF and starch concentrations as a result of the silages they were based on. The inclusion of a greater amount of corn meal, as a starch source, in the SSS and the SS diets to compensate these chemical differences might be the cause of the decrease in these saturated fatty acids (1).

Among MUFA, the amount of oleic acid (C18:1 cis-9) in cheese was higher from cows fed with the alternative silages (SSS and SS) than with corn silage (CS). This is highly desirable because oleic acid is considered to be hypocholesterolemic in humans. The higher amount of C18:1 n8 trans10 from cheese from the CS diet could be associated with the higher amount of dietary readily digestible carbohydrates present in corn silage.

The different types of silages used for dairy cows feeding did not modify the total PUFA content of cheese. This was mainly because the proportion of linoleic acid (C18:2 cis-9, cis-12) detected in cheeses were similar among the three different silages. But, the forage source was able to alter the distribution of specific CLA isomers because of the higher content of C18:2 cis9 trans11 CLA in cheese coming from the SS diet.

The n6:n3 ratio is an important human health index that should not >4 (35). Even though there were no significant differences among cheeses produced using diets based on these three types of silages, they all compiled with the recommended value which can be considered as relevant and desirable from a human nutritional point of view. The other two healthy indexes, atherogenic and thrombogenic, resulted favorable for the cheeses coming from the alternative crops rather than the corn silage diet because of the increase in the unsaturated FAs over the saturated FAs. Overall, the use of these two alternative silages had a good effect on cheese fatty acids profile as it does not enhance the risk of cardiovascular disease.

### 4.4 Cheese volatile profile

The volatile profile of cheese is considered one of the most important criteria for evaluating cheese quality. It is closely linked to the geographical area and the production methodology (36). The territory in which cows are housed and milked strongly influences the quality of the resulting cheese. In fact, cows' diet can affect the production and relative concentrations of various volatile compounds in both fresh and ripened cheeses (15). Traditional cheese-making methods, often preserved as cultural heritage and tightly connected to their place of origin, contribute to the remarkable diversity of cheeses found worldwide. The most common cheeses, especially those with Protected Designation of Origin (PDO) status, have been well characterized in terms of their volatile compound composition (37, 38). However, some traditional and regionally typical cheeses remain unknown. In the present study, the volatile profile of the Italian cheese Latteria is characterized for the first time and linked to diet of the cows. In general, the two main chemical classes found were alcohols and acids. The same categories characterized Montasio cheese at the same age of ripening (60 d) as the Latteria cheese in this study (36).

Cheese produced from cows fed with corn silage (CS) was located in the upper-right quadrant and characterized by the presence of several acids and esters. Among acids, medium-chain fatty acids (C_6_-C_12_) were the dominant constituents within this aromatic class and they are generally responsible for rind-like, goaty, sweat, fatty, and rancid sensory notes (37). The release of medium-chain fatty acids within the cheese matrix is largely attributed to the activity of lipoprotein lipase, an indigenous enzyme that remains active in raw-milk cheeses and hydrolyses triglycerides during ripening (39). Nevertheless, it cannot be excluded that some of the acids may also derive from the lipid metabolism of cheese microflora (40). Additionally, branched-chain fatty acids such as 3-methylbutanoic and 2-methylpropanoic acids, typically linked to sweaty and rancid notes, were also positively associated with the CS cheese, reflecting advanced proteolysis and conversion of amino acids into volatile fatty acids (41). Moreover, ethyl esters were dominant in the CS cheese, due to the high availability of free fatty acids that react with ethanol mainly derived from the fermentation of lactose or from amino acid catabolism (39). As reported in the literature, ethyl esters are commonly associated with sweet, fruity and floral notes (37).

Regarding cheese coming from the sorghum-soy silage diet (SSS), few aromatic compounds weakly described the cheese flavor, mainly alcohols such as ethanol, 1-butanol, 1-hexanol, 1-octanol, and straight-chain esters like ethyl acetate and ethyl butyrate. Primary alcohols derive from the reduction of aldehydes by alcohol dehydrogenases, thus conferring cheese some typical fruity and green notes (42). The SSS cheese was negatively correlated with ketones, which are common constituents of most dairy products.

Cheese produced using the sorghum silage diet (SS) was heterogeneously characterized by a wider range of volatile compounds, including ketones, secondary and aromatic alcohols, and short-chain fatty acids. Among ketones, acetoin and several 2-methyl ketones were positively associated to SS cheese. Acetoin, which comes from the metabolism of lactose and citrate, is generally responsible for sour milk notes. Compounds such as 2-heptanone and 2-nonanone, which belong to the family of 2-methyl ketones, are instead linked to fruity, floral, and musty aromatic notes (37). The presence of methyl ketones promotes also the formation of secondary alcohols, including 2-propanol, 2-pentanol, and 2-heptanol, previously identified as key odorants of several cheeses (43). Lastly, propionic and butyric acids played an important role in the flavor of the SS cheese, where the first has a typical vinegar smell, while the latter is typically linked to rancid odors and might derive from undesired butyric acid fermentation (42).

The PCA-based observations were further supported by the distribution of volatile compound classes illustrated in Figure 1. The CS cheese presented similar proportions of acids, esters, and alcohols showing a more balanced volatile composition than the SSS and SS cheeses. In contrast, SSS cheese was mainly composed by alcohols, in agreement with its weaker volatile profile and limited formation of secondary lipid- and amino acid–derived compounds. The SS cheese had the most heterogeneous volatile composition distribution, with a high proportion of short-chain acids, consistent with their broader array of metabolic pathways revealed by the PCA. Overall, these patterns highlight that cheeses coming from diets using three different silages developed a unique aromatic fingerprint, strongly shaped by the specific feeding treatment.

In conclusion, the use of different silages affected composition and volatile compounds of Latteria cheese. Cheese from cows fed corn silage clearly differed in physical properties and fatty acid profile from that of cows fed with alternative silages. Physical differences of cheese were marked in color (cheese from alternative silages being whiter) and more pronounced in hardness and elasticity (cheese from alternative silages being harder and less elastic). This outcome points toward a main effect of the type of silage in the diet, that could be due to the presence of tannins in sorghum silage. However, under practical conditions the effect of the diet is strongly correlated to the silage chemical composition, the starch source and the NDF content in the diet. An important result was that volatile compounds in cheese were clearly different based on the silage used in the diet. The possible distinction of Latteria cheese coming from different types of silages is interesting for consideration in the manufacture processing and in the preservation of this typical cheese in the market.

## Data Availability

The original contributions presented in the study are included in the article/supplementary material, further inquiries can be directed to the corresponding author.
